# Humoral Response to Hepatitis B and COVID-19 Vaccine among Maintenance Hemodialysis Patients

**DOI:** 10.3390/vaccines10101670

**Published:** 2022-10-07

**Authors:** Naomi Nacasch, Keren Cohen-Hagai, Nurit Tayar, Avraham Levian, Gloria Rashid, Sydney Benchetrit, Eran Neumark, Ori Wand, Tammy Hod, Yossi Rosman, Moshe Shashar, Ayelet Grupper, Pnina Shitrit

**Affiliations:** 1 Department of Nephrology and Hypertension, Meir Medical Center, Kfar Saba 4428163, Israel; 2 Sackler Faculty of Medicine, Tel Aviv University, Tel Aviv 6997543, Israel; 3 Clinical Laboratories, Meir Medical Center, Kfar Saba 4428163, Israel; 4 Department of Pulmonology, Barzilai Medical Center, Ashkelon 7830604, Israel; 5 Faculty of Health Sciences, Ben-Gurion University of the Negev, Beer-Sheva 8499000, Israel; 6 Renal Transplant Center and Nephrology Department, Sheba Medical Center, Ramat Gan 5262000, Israel; 7 Allergy and Clinical Immunology Unit, Meir Medical Center, Kfar Saba 4428163, Israel; 8 Department of Nephrology and Hypertension, Laniado Hospital, Netanya 4244916, Israel; 9 Department of Nephrology and Hypertension, Tel Aviv Sourasky Medical Center Tel Aviv, Tel Aviv 6423906, Israel; 10 Infection Control Unit, Meir Medical Center, Kfar Saba 4428163, Israel

**Keywords:** humoral response, hepatitis B vaccine, COVID-19 vaccine, hemodialysis

## Abstract

Maintenance hemodialysis (MHD) patients have impaired immunological responses to pathogens and vaccines. In this study, we compared the humoral response to HBV and COVID-19 vaccines in a cohort of MHD patients. Demographic and clinical characteristics of vaccine responders and non-responders were also compared, and the association between the humoral responses to both vaccines was evaluated. The cohort included 94 MHD patients who were vaccinated at least once for HBV and twice for COVID-19. Among the 94 patients, 28 (29.8%) did not develop protective titers to HBV. Hypertension, coronary heart disease, and heart failure were more common in non-responders. Among MHD patients, 85% had positive IgG anti-spike SARS-CoV-2 levels 6 months after two doses of BNT162b2 (Pfizer/Biotech) vaccine. Age and immunosuppressive therapy were the main predictors of humoral response to COVID-19 vaccine. We did not find any association between non-responders to HBV and non-responders to COVID-19 vaccine. There was no difference in IgG anti-spike titers between HBV responders and non-responders (505 ± 644 vs. 504 ± 781, *p* = 0.9) Our results suggest that reduced humoral response to hepatitis B is not associated with reduced response to COVID-19 vaccine. Different risk-factors were associated with poor immune response to HBV and to COVID-19 vaccines.

## 1. Introduction

Patients undergoing maintenance hemodialysis (MHD) are known to have impaired immunological responses to pathogens and vaccines. The uremic milieu, malnutrition and chronic inflammation in this population contribute to alterations in T lymphocytes and antigen-presenting cells, with consequently diminished humoral response [1,2,3,4]. Hence, MHD patients are at higher risk for acquiring infections, which continue to be a leading cause of death among patients with end-stage kidney disease [2].

The uremic milieu attenuates the differentiation and function of dendritic cells and compromises T cell functions, thus diminishing the immunologic response to vaccination [5,6,7]. Stachowski et al. showed that the addition of uremic serum to CD4 T-lymphocyte cultures from healthy individuals decreases T cell receptor (TCR) density on the CD4 T-lymphocyte surface; monocytes isolated from uremic patients have impaired MHC II expression after stimulation [8,9]. They also founded that TCR density on the T-lymphocyte surface correlates with the immune response to hepatitis B virus (HBV) vaccination in HD patients [10]. Thus, the decreased TCR quantity on the CD4 T-lymphocyte surface and the impaired MHC II expression on the monocyte surface may be responsible for the acquired immunity disturbances in dialysis patients; those could explain the diminished response to HBV vaccine as well as the disappointing results of vaccinations against other protein antigens in this population [5,11]. Data regarding HBV vaccination in patients on MHD showed reduced antibody response and lower antibody titers over time. Indeed, only 50–60% of dialysis patients develop protective titer of anti-hepatitis B antibodies after 3 doses of vaccine, as opposed to more than 90% in healthy individuals [3,4]. Older age and diabetes mellitus are known risk-factors for reduced vaccination response in both healthy individuals and dialysis patients [12,13,14]. In a recent meta-analysis, specific dialysis-related factors such as anemia, poor nutritional status, lower dialysis adequacy, lower parathyroid hormone levels and shorter time on dialysis were associated with reduced HBV vaccine response. Addressing some of these modifiable factors might improve vaccination response [15].

In late 2019, the Coronavirus disease 2019 (COVID-19) pandemic emerged. MHD patients are immunocompromised and may have other risk-factors associated with their primary renal disease, such as older age, diabetes, hypertension, etc. As such, they were found to be at increased risk for severe disease and mortality [16,17,18,19]. Therefore, with the release of the SARS-CoV-2 mRNA vaccine, MHD patients were prioritized for vaccination. The BNT 162b2 (Pfizer/Biotech) vaccine was one of the first approved vaccines worldwide. The mRNA vaccine initiated both the humoral and cellular immune responses, which correlates with protection from severe disease [20]. The two-dose vaccination regimen was found to reduce the occurrence of severe disease by over 90% [20]. However, emerging data on the response of MHD patients to the new vaccine showed reduced early humoral response as compared to healthy controls [21,22]. Danthu et al. showed that the early humoral response to the BNT162b2 mRNA vaccine is reduced in MHD patients and that non-responders to the HBV vaccine were also less likely to develop humoral response to the COVID-19 vaccine [23].

In this study, we aimed to evaluate the possible associations between the humoral response to HBV vaccine and late humoral response to the BNT162b2 mRNA vaccine among MHD patients. We hypothesized that the uremic milieu impairs the post-vaccine humoral response to both BNT162b2 mRNA and HBV vaccines and that common factors will contribute to diminished humoral response to the vaccines in this unique population.

## 2. Materials and Methods

This observational study was conducted at the hemodialysis unit of Meir Medical Center (MMC), Kfar Saba, Israel and included only patients with end-stage renal disease on maintenance hemodialysis, defined as least 3 months of hemodialysis. Meir Medical Center provides chronic dialysis treatment to 150 MHD patients. Results are reported according to STROBE statement guidelines.

### 2.1. Patients

MHD patients treated in the hemodialysis unit of Meir Medical Center were enrolled in the study. For each patient, demographic, clinical and laboratory data were recorded from the electronic medical records, including age, sex, comorbidities, primary renal disease, concomitant medications, dialysis vintage, complete blood count, blood chemistry, and dialysis adequacy parameters.

### 2.2. Anti-Hepatitis B Antibodies

All MHD patients in our center are routinely tested for HBV antigen and antibodies, as recommended by the Centers for Disease Control and clinical guidelines.

All patients were vaccinated against HBV virus at the dialysis unit at hemodialysis initiation if they had anti-hepatitis-B surface antibodies (HBsAb) < 10 mIU/mL and negative hepatitis-B surface antigen (HBsAg).

The HBV vaccine protocol in MMC dialysis unit includes administration of Sci-B-Vac™ vaccine (recombinant hepatitis B vaccine, manufactured by SciVac Ltd., Rehovot, Israel.), at 0, 1, and 6 months via 10 μg/mL intramuscular injection in each deltoid muscle (total 20 μg at each time point). HBsAb and HBsAg followed every 1 to 6 months, as appropriate.

HBV-vaccine response was defined as anti-HBs (HBsAb) titer ≥ 10 mIU/mL following immunization. Those with HBsAb < 10 mIU/mL were revaccinated. Data regarding hepatitis B vaccination dates and HBsAb titers were recorded.

### 2.3. SARS-CoV-2 Spike IgG

Patients received the first dose of BNT162b2 vaccine beginning December 2020 through January 2021 and the second dose 21 days after the first.

In July 2021, all patients were tested for antibodies to SARS-CoV-2 spike protein (IgG S) using the Abbot AdviseDx SARS-CoV-2 IgG II Quant assay on an Architect i200SR analyzer as part of another study [24]. A cutoff ≥ 50 AU/mL was considered a meaningful antibody response, as previously suggested [21,22]. Anti-nucleocapsid antibody (SARS-CoV-2) levels (anti-N Ab) were measured in all study participants to exclude asymptomatic covert infection. Anti-N-Ab were measured using the Architect SARS-CoV-2 IgG nucleocapsid protein assay (Abbot, Abbot Park, IL, USA) as previously described [25]. Patients with positive titers of anti-N Ab were excluded from this analysis.

### 2.4. Statistical Analysis

Descriptive statistics are presented as means with standard deviation and range, median, or percentage. Comparison of variables between two study groups was performed using *t*-test, Mann–Whitney test, Fisher’s exact test, or chi-square test according to the scale of the variables. *p*-values < 0.05 were considered statistically significant. A multivariate logistic regression model including all relevant variables was applied to estimate odds ratios (OR) for non-response to HBV vaccine. Data were analyzed with SPSS, Version 27 (IBM Corporation, Armonk, NY, USA).

### 2.5. Ethical Issues

The study was approved by the Ethics Committee and Institutional Review Board of Meir Medical Center (no. MMC-029-21) in July 2021. All study participants provided signed informed consent prior to enrollment. The study was performed in accordance with the Declaration of Helsinki and Good Clinical Practice guidelines.

## 3. Results

**Figure 1:**
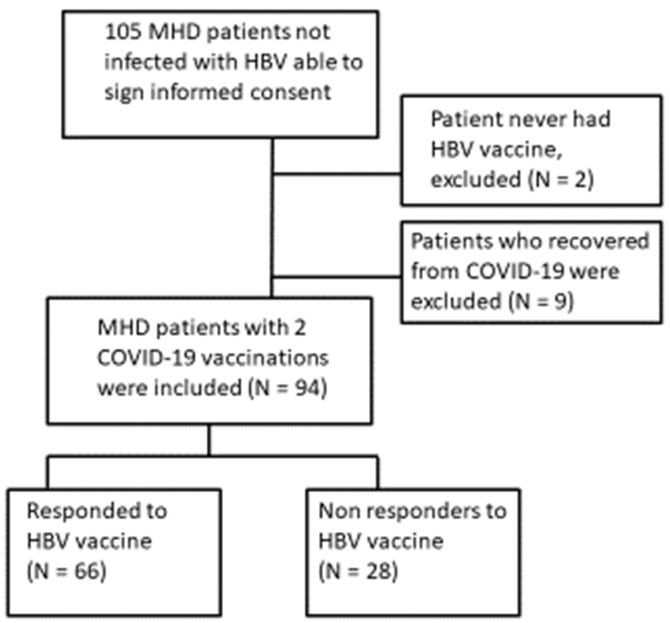
Study cohort.

The initial cohort included 105 MHD patients without previous exposure to HBV. Two patients were not vaccinated against HBV at all, and nine patients were infected with COVID-19 virus and were excluded from the analysis (Figure 1).

The final cohort included 94 MHD patients who were vaccinated at least once for HBV, and twice for COVID-19 with the BNT Pfizer vaccine.

HBsAb titer was <10 mIU/mL in 28/94 patients and ≥10 mIU/mL in 66. Therefore, we divided the cohort into 2 groups: HBV responders and HBV non-responders (Table 1). Hypertension, coronary heart disease, and heart failure were more prevalent among non-responders to the HBV vaccine.

### 3.1. SARS-CoV-2 Spike IgG Titers

IgG S titers were measured 6 months after the first BNT162b2 vaccine dose as described in our recent study examining the long-term antibody response to the BNT162b2 vaccine among MHD patients [24].

We found that age and immunosuppressive therapy were the main predictors of the humoral response to BNT162b2 among MHD patients 6 months following vaccination. Antibody levels were inversely correlated with age in our study, in agreement with other studies.

### 3.2. HBV Vaccine Response

Among the 94 study patients, 28 did not develop protective titers despite comparable time from last vaccine dose and comparable vaccine doses (4.8 ± 2.8 vs. 4.1 ± 1.8 vaccine doses, *p* = 0.183).

HBsAb titers significantly correlated with lymphocyte count (Spearman’s rho = 0.336, *p* = 0.005) and serum albumin level (Spearman’s rho = 0.237, *p* = 0.05) and inversely correlated with C-reactive protein levels (Spearman’s rho = −0.268, *p* = 0.03). There was a trend toward a correlation between dialysis adequacy (kt/v) and HBV titer (Spearman’s rho = 0.2, *p* = 0.06). A significant positive correlation between dialysis vintage (Spearman’s rho = 0.288, *p* = 0.005) and time from HBV vaccine (Spearman’s rho = 0.439, *p* = 0.0001) was found. Further correlation results are detailed in Table 2. In a multivariate regression analysis model, coronary heart disease and heart failure were significant predictors of no response to HBV vaccine (Table 3).

### 3.3. HBV and SARS-CoV-2 IgGs Titers

IgG S titers were measured 6 months after the first vaccine dose as described in our study regarding long-term antibody response to the BNT162b2 vaccine among maintenance hemodialysis patients [24]. Time from last HBV and COVID-19 vaccine was comparable between groups (*p* = 0.45 and 0.55, respectively).

There was no difference in IgG S titers between HBV responders and non-responders (505 ± 644 vs. 504 ± 781, *p =* 0.9) and in non-responders to COVID-19 vaccine (19.7% vs. 14.3%, *p* = 0.5; Table 1).

**Figure 2:**
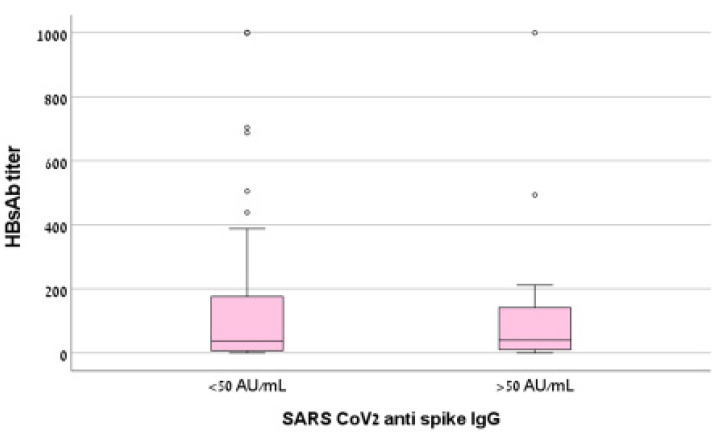
Box plot of HBsAb titer (mIU/mL) according to humoral response to BNT Pfizer vaccine. IgG S titer was not correlated with HBV titer (Spearman’s rho = 0.148, *p* = 0.155). HBV: hepatitis B virus; HbsAb: hepatitis B surface antibody.

Mean HBsAb titer was 142.9 ± 253.7 mIU/mL in patients with low antibody response to SARS-CoV-2 vaccine and 180.2 ± 300.1 mIU/mL among patients with >50 antibody response (*p* = 0.635). Median levels are shown in Figure 2.

## 4. Discussion

This study evaluated the long-term humoral response to HBV and COVID-19 vaccines among MHD patients and the factors associated with poor response to these vaccines. In this cohort of MHD patients, 30% did not develop an antibody response to HBV vaccine, while only 15% did not have a response to the BNT162b2 Pfizer vaccine 6 months after COVID-19 vaccination. These findings are in agreement with other papers evaluating antibody response to HBV vaccine in MHD patients [14,26].

Contrary to our expectations, we found no correlation between immune responsiveness to HBV and COVID-19 vaccines. IgG S titers to COVID-19 vaccine were comparable between HBV vaccine responders and non-responders. Poor response to COVID-19 vaccine did not predict poor response to HBV vaccine. Furthermore, factors for poor response to vaccine were different between the vaccines.

The prevalence of HBV vaccine responders (70%) in our cohort was comparable to that described in previous cohorts of MHD patients [1,4]. In our cohort, coronary heart disease and heart failure were more common among HBV non-responders and were the most important predictors of poor response to HBV vaccine in a multivariate regression analysis model that included other comorbidities and poor response to COVID-19 vaccine.

A correlation test in our cohort demonstrated significant associations between HBsAb titers and malnutrition inflammation markers. We observed a significant positive correlation between immune response to HBV and serum albumin and inverse correlation to C-reactive protein. Absolute lymphocyte count and dialysis adequacy were also positively correlated with HBsAb titers. These findings, in addition to the growing evidence that nutritional status has a fundamental role in the outcome of MHD patients, specifically suggest a role in the complex immune response to vaccine [1,27,28,29]. This finding has clinical significance. Malnutrition is a common phenomenon among MHD patients and is potentially reversible with various treatment options.

As previously reported by our group and others, poor humoral response to the BNT162b2 COVID-19 vaccine in MHD patients was associated with older age and immunosuppressive therapy [20,21,22,23,24].

Our findings are in contrast to those of Danthu et al., who found lower immune response to COVID-19 among MHD patients with the lowest antibody titers to HBV vaccine [23]. The timing of testing the humoral response to the COVID-19 vaccine differed, which may obscure an association. We focused on the long-term immune response 6 months following the first vaccine dose and measured IgG titers, whereas Danthu et al. analyzed IgG titers 14 to 58 days following vaccine administration [23]. Of note, we chose the cutoff of ≥50 AU/mL based on previous publications. We realize that this is not a well-established cutoff and that the correlation between given antibody level after the BNT162b2 vaccine and actual protection is not clear enough. Neutralization assay or cellular assays will be considered in our future studies in order to improve the understanding of the impact of the immune response in hemodialysis patients. However, we did evaluate COVID-19 infection, and published the data separately [30], as well as the possible impact of different dialysis modality on humoral response to BNT162b2 vaccine [31].

Our findings emphasize the multi-factorial nature and the complexity of the humoral response to vaccine and demonstrate that the clinical and humoral responses to different vaccines are not necessarily uniform. In our study, 85% of MHD patients developed a seropositive response to the BNT162b2 vaccine as compared to a 70% response rate to the Sci-B-Vac™ vaccine. It was suggested that the differing response rates might be due to the different natures of the vaccines. mRNA vaccine could increase immunological response via induction of type I interferon expression in dendritic cells [32,33,34]. This should prompt further investigations evaluating immune response to vaccines among MHD patients and influencing reversible factors.

This study had some limitations. First, it was a relatively small, single-center study, and as such, the findings should promote further research in this field rather than be generalized to other populations. We assessed humoral response at one point only, with no data immediately post-vaccination. Nevertheless, given the data published by others [22,23], we can assume that the IgG S titer was even higher shortly after vaccination. Data published by Danthu et al. were from measurements taken not only at different time points but also using a different assay with a different cutoff for assessing the humoral response of dialysis patients to the Pfizer/BioNTech (BNT162b2) vaccine. Therefore, comparison should be made carefully. However, we believe limited conclusions can be drawn from this analysis given the differences in immunogenicity based on the vaccine platform as well as the lack of a defined correlation of protection for SARS-CoV-2 with an arbitrary cutoff selected.

However, long-term data regarding the humoral response are strongly needed, and data from a single center with a heterogeneous population, such as MHD patients, may contribute to the understanding of the complex, multi-factorial, diminished humoral response in the MHD population.

## 5. Conclusions

In conclusion, hemodialysis-dependent patients are at risk for decreased humoral response to vaccines against several preventable diseases due to alterations in their immune system as well as other common risk factors, such as older age, comorbidities, and immunosuppressive therapies. In our cohort, responsiveness to HBV vaccine was not associated with diminished humoral response to another vaccine (COVID-19) and risk factors associated with appropriate immune response to the vaccine differed. Further research evaluating immune response to vaccines in the MHD population are needed.

## Figures and Tables

**Table 1:** Clinical, demographic and baseline laboratory data of study cohort by response to HBV vaccine.

Variable	HbsAb < 10 mIU/mL (*n* = 28)	HbsAb ≥ 10 mIU/mL (*n* = 66)	*p*-Value
Age (years)	72.1 ± 12.2	69.8 ± 15.5	0.5
Weight (kg)	82.1 ± 26.6	75.9 ± 16.2	0.2
Sex (male)	18 (64.3)	44 (66.7)	0.8
IGG S titer (AU/mL)	504 ± 781	505 ± 644	0.9
IGG S < 50 (AU/mL)	4 (14.3)	13 (19.7)	0.5
Diabetes mellitus	21 (75)	40 (60.6)	0.2
Hypertension	27 (96.4)	48 (72.7)	0.01
Coronary heart disease	19 (67.9)	15 (22.7)	<0.001
Heart failure	17 (60.7)	15 (22.7)	<0.001
Peripheral vascular disease	3 (10.7)	7 (10.6)	0.9
Malignancy	0	1 (1.5)	0.5
Chronic immunosuppressive therapy	1 (3.6)	4 (6.1)	0.6
kt/V	1.21 ± 0.22	1.31 ± 0.22	0.07
Urea reduction ratio	64.9 ± 7	67.5 ± 6.5	0.1
Dialysis vintage (months)	20.6 ± 16.4	34.8 ± 25.7	0.008
White blood cells (K/µL)	6 ± 2	6.5 ± 2	0.4
Lymphocytes (K/µL)	1 ± 0.4	1.4 ± 0.6	0.03
Platelets (K/µL)	169 ± 43	195 ± 71	0.2
Creatinine (mg/dL)	6.8 ± 2.4	7.5 ± 2.4	0.3
Albumin (gr/dL)	3.6 ± 0.3	3.6 ± 0.5	0.5
C-reactive protein (mg/dL)	2.2 ± 1.9	2 ± 3.6	0.9
PTH (pg/mL)	359 ± 296	290 ± 215	0.3

HBV: hepatitis B virus; HbsAb: hepatitis B surface antibody (mIU/mL); IgG S: SARS-CoV-2 spike protein antibodies (AU/mL); PTH: parathyroid hormone; kt/V: a value used to quantify hemodialysis adequacy; k: dialyzer clearance of urea; t: dialysis time; v: volume of distribution of urea.

**Table 2:** Variables that were correlated with HBsAb titers.

Variable	Correlation Coeffiecient (Spearman’s Rho)	*p*-Value
Age (years)	−0.095	0.4
Weight (kg)	−0.006	0.9
IGG S titer (AU/mL)	0.15	0.2
kt/V	0.2	0.06
Dialysis vintage	0.3	0.005
White blood cells (K/µL)	0.1	0.3
Lymphocytes (K/µL)	0.3	0.005
Albumin (gr/dL)	0.2	0.05
C-reactive protein (mg/dL)	−0.3	0.03
PTH (pg/mL)	−0.03	0.8

HbsAb: hepatitis B surface antibody; IgG S: SARS-CoV-2 spike protein antibodies; PTH: parathyroid hormone.

**Table 3:** Multivariate regression analysis for predictors for no response to HBV vaccine.

Variable	Odds Ratio	95% Confidence Interval		*p*-Value
		Lower	Upper	
Poor response to COVID-19 vaccine	0.52	0.11	2.40	0.40
Sex (female)	1.55	0.50	4.87	0.45
Immunosuppressive therapy	1.19	0.06	23.82	0.91
Diabetes mellitus	1.06	0.31	3.63	0.92
Hypertension	3.13	0.34	28.89	0.31
Coronary heart disease	7.06	2.18	22.87	0.00
Heart failure	4.22	1.41	12.64	0.01
Malignancy	0.00			1.00

HBV: hepatitis B virus.

## Data Availability

Data are available on request from the corresponding author.
